# Identification of Fatty Acid Desaturase 6 in Golden Pompano *Trachinotus Ovatus* (Linnaeus 1758) and Its Regulation by the PPARαb Transcription Factor

**DOI:** 10.3390/ijms20010023

**Published:** 2018-12-21

**Authors:** Ke-Cheng Zhu, Ling Song, Hua-Yang Guo, Liang Guo, Nan Zhang, Bao-Suo Liu, Shi-Gui Jiang, Dian-Chang Zhang

**Affiliations:** 1 Key Laboratory of South China Sea Fishery Resources Exploitation and Utilization, Ministry of Agriculture and Rural Affairs, South China Sea Fisheries Research Institute, Chinese Academy of Fishery Sciences, 231 Xingang Road West, Haizhu District, Guangzhou 510300, China; zkc537@163.com (K.-C.Z.); 18620160853@163.com (L.S.); guohuayang198768@163.com (H.-Y.G.); zsdxgl@163.com (L.G.); 398730316@163.com (N.Z.); liubaosuo343@163.com (B.-S.L.); jiangsg@21cn.com (S.-G.J.); 2 Engineer Technology Research Center of Marine Biological Seed of Guangdong Province, Guangzhou 510300, China; 3 Key Laboratory of Fishery Ecology & Environment, South China Sea Fisheries Research Institute, Guangzhou 510300, China; 4 South China Sea Bio-Resource Exploitation and Utilization Collaborative Innovation-Center, South China Sea Fisheries Research Institute, Guangzhou 510300, China

**Keywords:** *Trachinotus ovatus*, fatty acid desaturases, PPARαb and Fads6, transcriptional activity, heterologous expression

## Abstract

Fatty acid desaturases are rate-limiting enzymes in long-chain polyunsaturated fatty acid biosynthesis. The transcription factor peroxisome proliferator-activated receptor alpha b (PPARαb) regulates lipid metabolism in mammals, however, the mechanism whereby PPARαb regulates fatty acid desaturases is largely unknown in fish. In this study, we report the full length cDNA sequence of *Trachinotus ovatus* fatty acid desaturase, which encodes a 380 amino acid polypeptide, possessing three characteristic histidine domains. Phylogenetic and gene exon/intron structure analyses showed typical phylogeny: the *T. ovatus* fatty acid desaturase contained a highly conserved exon/intron architecture. Moreover, functional characterization by heterologous expression in yeast indicated that *T. ovatus* desaturase was a fatty acid desaturase, with Δ4/Δ5/Δ8 Fad activity. Promoter activity assays indicated that *ToFads6* desaturase transcription was positively regulated by PPARαb. Similarly, PPARαb RNA interference decreased ToPPARαb and ToFads6 expression at the mRNA and protein levels in a time-dependent manner. Mutation analyses showed that the M2 binding site of PPARαb was functionally important for protein binding, and transcriptional activity of the *ToFads6* promoter was significantly decreased after targeted mutation of M2. Electrophoretic mobile shift assays confirmed that PPARαb interacted with the binding site of the *ToFads6* promoter region, to regulate *ToFads6* transcription. In summary, PPARαb played a vital role in *ToFads6* regulation and may promote the biosynthesis of long-chain polyunsaturated fatty acids by regulating *ToFads6* expression.

## 1. Introduction

Long-chain polyunsaturated fatty acids (LC-PUFA) are involved in numerous biological processes and are major components of complex lipid molecules [1]. In vertebrates, two LC-PUFA biosynthetic pathways are defined: the “∆6 pathway” (∆6 desaturation-elongation-∆5 desaturation) and the “∆8 pathway” (elongation-∆8 desaturation-∆5 desaturation), which are initiated from α-linolenic (C18:3n-3) and linoleic (C18:2n-6) acids, respectively [1,2,3,4,5]. Two sets of enzymes, the elongation of very long-chain fatty acids (Elovl) and fatty acyl desaturases (Fads), are involved in these pathways [6]. The Fads protein family include Fads1–6; however, only Fads2 and Fads6 have been characterized in vertebrate. The Fads2 enzyme has been widely studied in teleosts, especially in marine species [7,8,9,10,11]. Previous studies using yeast heterologous expression systems have indicated that Fads2 exhibits ∆6, ∆5, ∆4, and ∆8 activity in the biosynthetic pathway of LC-PUFAs [1,4,7,8,9,10,11]; however, little information on *Fads6* has been reported. Ngoh et al. (2015) [12] revealed that fads6 expression was detected to be underlying, and suitable as a bioindicator to identify wild and farmed *Lates calcarifer* [1,2]. Some studies stated that *Fads6* is involved with lipid and fatty acid metabolism in *Homo sapiens* [13,14]. Moreover, *Fads6* displayed Fad synthesizing activity, but not Fad hydrolase activity, an activity which was localized to the N-terminal domain of full length Fads1 or 2 in *H. sapiens* [15]. However, the function of marine fish *Fads6* appears ambiguous during LC-PUFA biosynthesis. It is also unclear whether the *Fads6* gene possesses analogous fatty acid synthesis functions, as *Fads2* in marine fish.

The peroxisome proliferator-activated receptor alpha (PPARα) is a member of the steroid receptor superfamily of ligand-activated nuclear transcription factors, and is known to regulate lipid and glucose metabolism [16]. Lipid metabolism, including the inhibition of lipogenesis and the activation of fatty acid oxidation, is regulated by PUFA and their metabolites, along with transcription factors such as PPAR, hepatocyte nuclear factor-4 (HNF4), nuclear receptor subfamily 1 group H member 3 (NR1H3), sterol-regulatory element binding protein (SREBP), and nuclear factor-kappa B (NF-κ B) [17]. PPARα stimulates the expression of target genes, through direct binding to PPAR response elements (PPREs) in the promoter region of target genes [18,19]. Tang et al. [20] showed that PPARα bound to the Δ6 desaturase promoter region significantly enhanced the transcription of Δ6 Fad in human hepatocytes (HepG2 cells). It was also shown that PPARα upregulated *Fads2* promoter activity in fish and avians [21,22]. Both PPARα1 and PPARα2 activated the promoter activity of *Fads2* in *Latbrax japonicus*, but no such regulatory activity was detected for *Pseudosciaena crocea* [21]. Moreover, it was suggested that PPARγ was a trans-acting factor that promoted ∆6/∆5 desaturase expression in the marine teleost *Siganus canaliculatus* [23]. Nevertheless, the role of PPARα in the positive regulation of *Fads6* remain largely unknown in fish.

The golden pompano *Trachinotus ovatus* (Linnaeus 1758), Carangidae, and Perciformes are found in the Asia-Pacific region and are important aquaculture fish in China due to their economic value [24,25]. *T. ovatus* muscle was found to be rich in PUFAs [26], meaning it is an ample source of endogenous PUFAs. Consequently, *T. ovatus* is an exceptional model in which to investigate LC-PUFA biosynthesis regulatory mechanisms. To investigate the underlying function of *ToFads6* and the transcriptional regulation of PPARα during LC-PUFA biosynthesis, this study sought to clarify the importance of PPARαb in regulating *Fads6* transcriptional activity. First, a functional characterization of the *ToFads6* gene was performed using heterologous expression in yeast. Second, promoter activity assays were performed via the mutation of potential PPARαb binding sites to identify key element in the *Tofads6* promoter. Third, the overexpression (agonist) and suppression of expression (RNAi and inhibitors) of PPARαb was used to elucidate the transcriptional regulation of PPARαb, with respect to *ToFads6*. Last, the role of the PPARαb M2 binding site in the *Tofads6* promoter was investigated using the electrophoretic mobility shift assay (EMSA). These approaches helped identify *Fad6* function in marine fish, and showed that PPARαb performed a vital function in *Fads6* expression regulation.

## 2. Results

### 2.1. Molecular Cloning and Phylogenetics of T. ovatus Fads6

The *T. ovatus* putative desaturase full length cDNA was 1594 bp in length and included an ORF of 1143 bp. This nucleotide sequence translated to a peptide sequence of 380 amino acids (accession no. MG674450) (Figure S1). A Basic Local Alignment Search Tool (BLAST) analysis revealed that the ToFads6 protein sequence shared high sequence identity with Fads6 sequences from other teleosts, including the tilapia (*Oreochromis niloticus*, 85%), stickleback (*Gasterosteus aculeatus*, 78%), fugu (*Takifugu rubripes*, 76%), and zebrafish (*Danio rerio*, 74%), and low sequence identity with humans (*Homo sapiens*, 58%).

**Figure 1:**
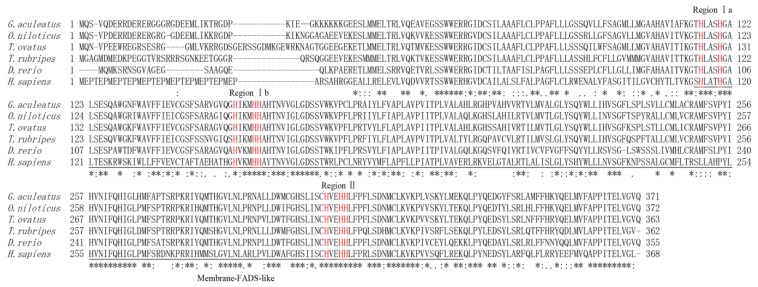
Amino acid sequences of *Trachinotus ovatus* Fads6 and with other homologs. Sequences comparisons revealed three regions of conserved histidine (his) cluster motifs containing eight his residues sites: HXXXH, HXXXHH, and HXXHH. Eight conserved his residues sites which are putative di-iron ligands are marked in red. Membrane-FADS-like domains are underlined. Accession numbers of Fads6 sequences are listed in Table S3. Identical and similar residues are marked with ‘*’ and ‘:’, respectively.

**Figure 2:**
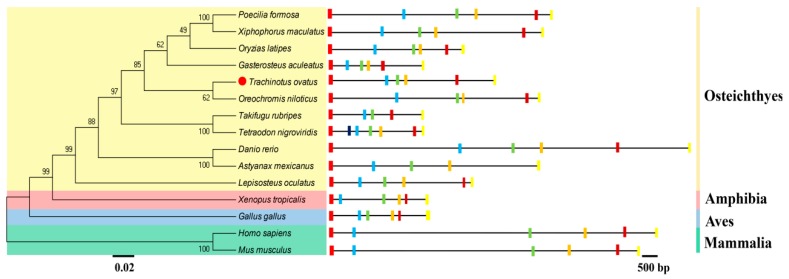
Genome structure analysis of *ToFads6* genes according to the phylogenetic relationship. Exon and intron lengths of each *PPARα* gene are displayed proportionally. Different color boxes and lines represent exons and introns, respectively. Identical color boxes represent homologous sequences.

Interestingly, comparisons of amino acid sequences for the Fads6 protein from these six species, revealed a membrane-Fads-like domain and three regions (Ia, Ib, and II) of conserved primary sequence, containing histidine-rich HX_3_H, HX_3_HH, and HX_2_HH motifs (Figure 1). Phylogenetic tree analysis indicated that ToFads6 clustered with several other Fads6 sequences from other osteichthyes, and more distantly, with amphibian, avian, and mammalian Fads6 (Figure 2). ToFads6 was grouped together with perciformes, such as *O. niloticus*. The distributions and length of exons and introns from each *Fads6* gene are shown in Table S2. All *Fads6* proteins contained six exons and five introns for all metazoans, except for the *Tetraodon nigroviridis Fads6*, which contained seven exons, while the *Astyanax mexicanus Fads6* contained five exons. Furthermore, the sizes of homologous introns sequences were different, while homologous exons sequences had almost no diversity.

### 2.2. Heterologous Expression of the Desaturase ORF in Saccharomyces cerevisiae

**Figure 3:**
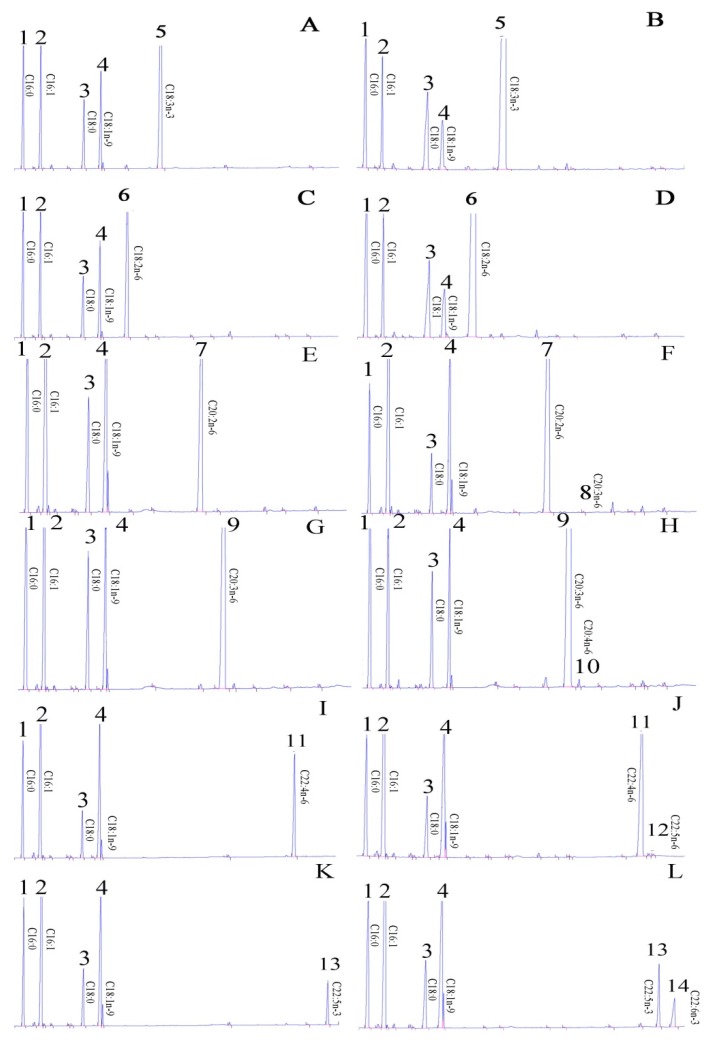
Functional characterization of the putative *ToFads6* in transgenic yeast. Fatty acid methyl esters (FAMEs) are extracted from yeast transformed with pYES2-Fads6, and grown in the presence of PUFA substrates C18:3n-3 (**A**), C18:2n-6 (**C**), C20:2n-6 (**E**), C20:3n-6 (**G**), C22:4n-6 (**I**), and C22:5n-3 (**K**). All left panels are yeast cells with empty vector. Based on retention times, additional peaks were identified as C18:3n-3 (**B**), C18:2n-6 (**D**), C20:3n-6 (**F**), C20:4n-6 (**H**), C22:5n-6 (**J**), and C22:6n-3 (**L**). Peaks 1–4 represent the main endogenous FAs in yeast, namely C16:0, C16:1 isomers, C18:0, and C18:1n-9, respectively. Moreover, peaks 5–14 represent exogenously supplemented FAs and the corresponding desaturation products, including C18:3n-3 (5), C18:2n-6 (6), C20:2n-6 (7), C20:3n-6 (8), C20:3n-6 (9), C20:4n-6 (10), C22:4n-6 (11), C22:5n-6 (12), C22:5n-3 (13), and C22:6n-3 (14), respectively.

The function of ToFads6 was characterized by determining FA profiles in *S. cerevisiae*, transformed with pYES2-Fads6, and grown in the presence of potential FA substrates, such as C18:2n-6, C18:3n-3, C20:2n-6, C20:3n-6, C22:5n-3, and C22:4n-6. In yeast transformed with pYES2-Fads6 and grown in the presence of C20:2n-6 (peak 7) (Figure 3E), C20:3n-6, (peak 9) (Figure 3G), C22:4n-6, (peak 11) (Figure 3I), and C22:5n-3 (peak 13) (Figure 3K) however, additional FA peaks were identified as C20:3n-6 (peak 8) (Figure 3F), C20:4n-6 (peak 10) (Figure 3H), C22:5n-6 (peak 12) (Figure 3J), and C22:6n-3 (peak 14) (Figure 3L), based on gas chromatography (GC) retention times. From this data, it was concluded that the ToFads6 possessed Δ4/Δ5/Δ8 Fad activity. The conversion rates of C20:2n-6 to C20:3n-6, C20:3n-6 to C20:4n-6, C22:4n-6 to C22:5n-6, C22:5n-3, to C22:6n-3 were calculated to be approximately 0.17%, 0.54%, 1.04%, and 39.29%, respectively (Table 1). Moreover, by using the gas mass spectrometry database, our results indicated that the C18:3n-6 and C18:4n-3 mass spectrometry structures were not detected; however, C20:3n-6, C20:4n-6, C22:5n-6, and C22:6n-3 structures were observed (Figure S2). Additionally, this experiment showed ToFads6 had no Δ6 desaturase activity, as the yeast expressing the *T. ovatus* desaturase was unable to desaturate FA substrates, such as C18:2n-6 and C18:3n-3 (Table 1; Figure 3A–D).

### 2.3. Tissue Distribution of ToFads6

**Figure 4:**
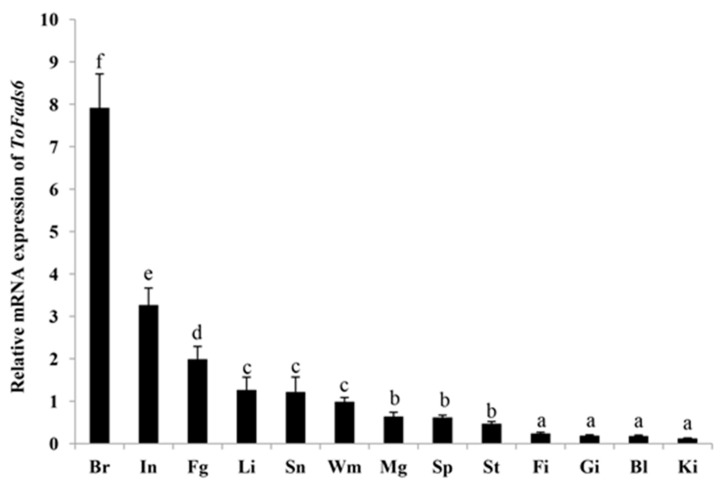
Gene transcriptions of *ToFads6* in various tissues. The tissues are small intestine (In), head-kidney (Ki), white muscle (Wm), stomach (St), female gonad (Fg), male gonad (Mg), brain (Br), liver (Li), gill (Gi), spleen (Sp), fin (Fi), blood (Bl), and snout (Sn). Different letters refer to significant differences.

Tissue distributions of *ToFads6* were delineated by qRT-PCR. The highest *ToFads6* mRNA levels were detected in the brain, followed by the small intestine and the female gonads and relatively low *ToFads6* expression levels were observed in fin, gill, blood, and kidney (Figure 4). Notably, the expression of *ToFads6* in the brain was much higher than in other tissues (*p* < 0.05).

### 2.4. Promoter Analysis of PPARαb Regulation

**Figure 5:**
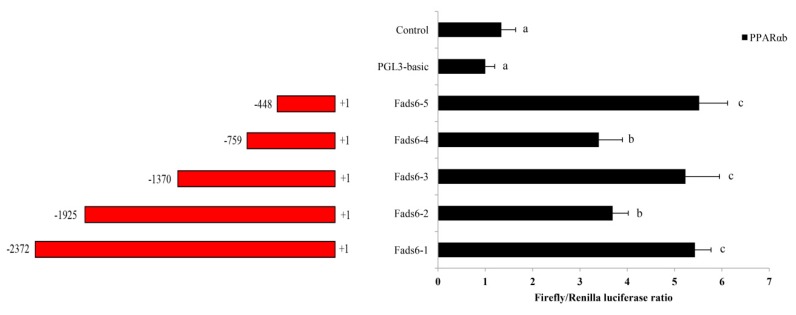
Promoter activity analysis of *ToFads6*. pGL3-Basic-Fads6s were cotransfected with the transcription factor PPARαb into 293 T cells. All values are presented as the mean ± SD (*n* = 3). Asterisks indicate different values, with respect to controls (* *p* < 0.05 and ** *p* < 0.01). Bars on the same group with different letters are statistically significant from one another.

**Figure 6:**
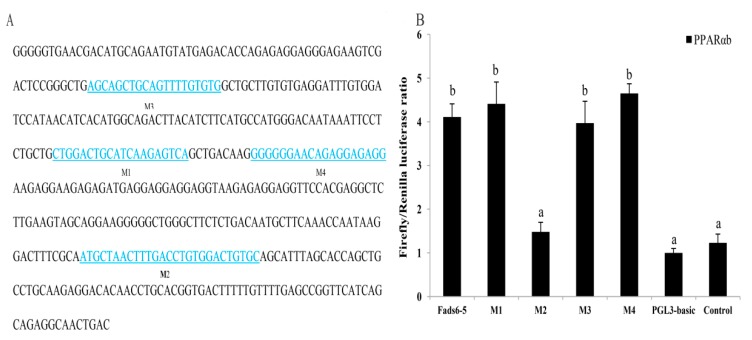
The nucleotide sequence and predicted binding sites for transcription factors in the core *ToFads6* promoter region (**A**). Effects of transcription factor mutations on *ToFads6-5* promoter activity (**B**). Binding sites are shown with boxes M1–M4. Mutations in promoter sequences are listed in Table 2. All values are presented as the mean ± SD (*n* = 3). Asterisks indicate values are different from controls (* *p* < 0.05 and ** *p* < 0.01). Bars on the same group with different letters are statistically significant from one another.

The cloned candidate *ToFads6* promoter (2372 bp) was an upstream nontranscribed sequence. To determine the binding region of PPARαb in the *ToFads6* promoter, a full-length candidate promoter and several truncated mutants were constructed with a promoter-less luciferase reporter vector, pGL3-basic. The promoter construct, Fads6-p5 (−448 bp to +1 bp) showed the highest promoter activity with PPARαb, suggesting that this region of the Fads6-p5 promoter sequence contained the PPARαb binding site (Figure 5). To identify the PPARαb binding sites in the *ToFads6* promoter, the predicted binding sites were mutated (Figure 6A, Table 2). The effects on promoter activity were investigated in 293T cells, transfected with each mutant and PPARαb. The results revealed that mutation of the M2 binding site (−113 bp to −87 bp) caused significant reduction in promoter activity (Figure 6B), showing that M2 was the PPARαb binding site in the *ToFads6* promoter. Notably, three other predicted binding sites did not induce luciferase activity with PPARαb, suggesting that these three sites were not required for triggering *ToFads6* expression with PPARαb.

### 2.5. Transcriptional Regulation of ToFads6 by PPARαb

**Figure 7:**
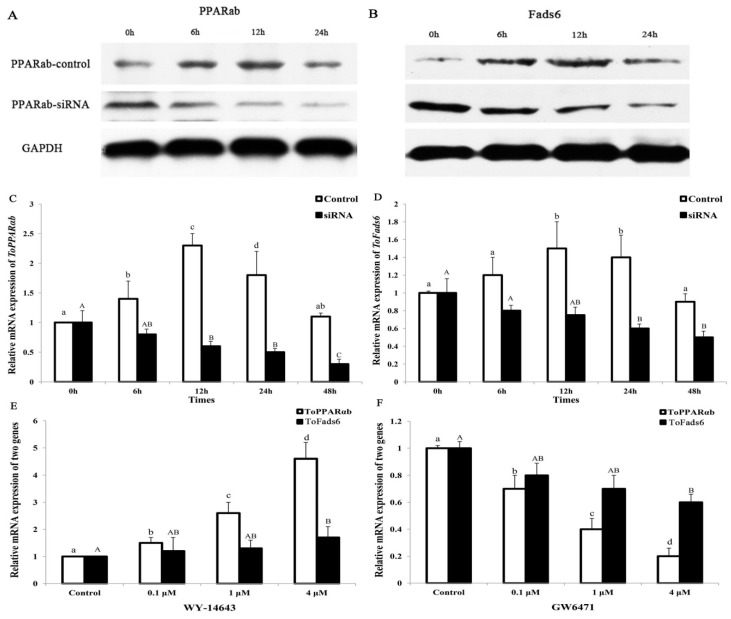
*ToPPARαb* upregulates *ToFads6* expression. Western blot and real-time PCR analysis were used to detect *ToPPARαb* expression (**A**,**C**) and *ToFads6* expression (**B**,**D**) after the transfection of either control RNA (control) or siRNA (RNAi), respectively. TOCF cells were stimulated with 0.1, 1, and 4 mM of PPARαb agonist (WY-14643) (**E**) and inhibition (GW6471) (**F**) for 24 h. The expression of *ToPPARαb* and *ToFads6* was dramatically increased and decreased in a concentration-dependent manner, respectively. ToPPARαb expressions were described as Zhu et al. [27]. All values were expressed as the mean ± SD (*n* = 3). Bars on the same group with different letters are statistically significant from one another (*p* < 0.05).

Protein and mRNA levels of *ToPPARαb* were significantly decreased in a time-dependent manner by RNAi knockdown of *PPARαb*, suggesting effective knockdown of *ToPPARαb* expression in *T. ovatus* caudal fin (TOCF) cells (Figure 7A,C) [27]. When *ToPPARαb* expression was reduced, protein and mRNA levels of *ToFads6* were dramatically depleted, when compared with the control group at the corresponding time points (Figure 7B,D). Moreover, PPARαb-mediated agonist activity (WY-14643) dramatically increased *ToFads6* mRNA levels (*p* < 0.05) (Figure 7E), and similarly the expression of *ToFads6* was dramatically suppressed upon addition of the PPARαb inhibitor, GW6471 in a concentration-dependent manner, over a 24 h stimulation period (Figure 7F). These results suggested an active regulatory role of *ToPPARαb* on *ToFads6* expression in TOCF cells.

### 2.6. Binding of PPARαb to Fads6 Promoters

**Figure 8:**
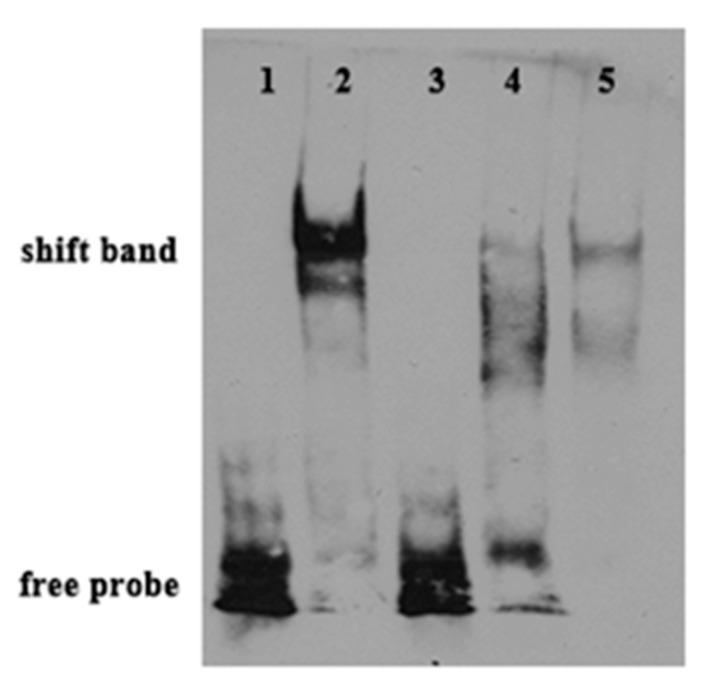
Binding reactions of ToPPARαb and *ToFads6* promoters. Biotin-labeled electrophoretic mobility shift assay (EMSA) probes were incubated with HEK293T lysates containing recombinant PPARαb protein. WT, wild-type probe; MT: mutated probe. 1, Fads6-WT; 2, Fads6-WT+Protein; 3, Fads6-WT+Flag; 4, Fads6-WT (cold 100×) + Protein; 5, Fads6-MT + protein.

To confirm the PPARαb binding motif in the *ToFads6* promoter, an EMSA assay was performed. Based on the predicted ToPPARαb binding site, oligonucleotide probes were synthesized and incubated with HEK293T cell lysates, including recombinant PPARαb in vitro. Recombinant PPARαb bound to the oligonucleotide probes of the predicted PPARαb binding site in the *ToFads6* promoter; however, mutations in the PPARαb binding site (Table S1), resulted in the dissociation of the DNA-rPPARαb complex (Figure 8), suggesting that PPARαb was specifically interacting with the M2 motif in the *ToFads6* promoter. The formation of a DNA-rPPARαb complex was specific, since it could only be blocked by excessive unlabeled control probes (100×).

## 3. Discussion

This study has generated insights into mechanisms underlying the transcriptional regulation of LC-PUFA biosynthesis in *T. ovatus*. To achieve this, the sequence and functional characterization, tissue expression patterns, and transcriptional regulation of *ToFads6* were investigated. The *ToFads6* ORF encoded a protein that was 74–85% identical to Fads6 proteins from other teleosts. These proteins contained three classical histidine-rich structural motifs: (i) HX_3_H, (ii) HX_3_HH, and (iii) HX_2_HH boxes, which are present in membrane-bound desaturase domains. These three conserved histidine-rich boxes are also present in other Fads2 proteins from other species, and are believed to be essential for binding iron ligands, for enzyme activity [11,28,29,30,31]. Phylogenetic analysis showed a typical phylogeny, revealing the amino acid sequence of ToFads6 closely matched the Fads6 of *O. niloticus*, but then appeared to separate from other fish, amphibians, avian and mammalian species. A genome structure analysis indicated that all *Fads6s* contained six exons and five introns in metazoans, except for *Fads6* from *T. nigroviridis* and *A. mexicanus*. These observations may represent intron gain or loss during evolutionary processes [32].

Two members of the Fads protein family have been described in teleosts: Fads2 and Fads6. For marine fish, yeast heterologous expression systems indicated that *Fads2* showed Δ4/5/6/8 activity in LC-PUFA biosynthesis [1,4,7,8,9,10,11], however, no information was available on the underlying function of *Fads6*. In this study, the functional characteristics of *T. ovatus Fads6*, via heterologous expression in *S. cerevisiae,* indicated that the *T. ovatus* putative desaturase was *Fads6*, with Δ4 desaturation activity. This was functionally similar to the *Fads6* from *Arabidopsis thaliana*, *Medicago truncatula*, and *Homo sapiens* [12,13,14]. *T. ovatus Fads6* exhibited low activity towards 22:4n-6 and 22:5n-3 substrates, such as 22:5n-6 (1.04%) and 22:6n-3 (39.29%). These results were similar to freshwater and marine fish, such as 10.2% and 3.6% in *Channa striata* [33], 14.0% and 23.0% in *Siganus canaliculatus* [8], 16.1% and 6.8% in *Solea senegalensis* [34], 13.7% and 24.1% in *O. latipes*, and 8.1% and 10.8% in *O. niloticus*, for *Fads2* with Δ4 desaturation activity [35]. It was also suggested that an equivalent highly unsaturated fatty acid (HUFA) synthesis activity of *ToFads6* was observed in other teleosts *Fads2*, which exhibited Δ4 desaturation activity. Nevertheless, teleost *Fads2* did not exhibit the above functions in the biosynthesis of saturated LC-PUFA [29,36,37]. Two LC-PUFA biosynthesis pathways from α-linolenic (C18:3n-3) and linoleic (C18:2n-6) acids have been proposed for *T. ovatus* [4,8,35].

In this study, the highest *ToFads6* mRNA expression was detected in the brain, showing that essential fatty acid metabolism occurs there [38]. However, relatively moderate *ToFads6* mRNA expression levels were detected in the intestine and liver. Interestingly, these are the first tissues exposed to dietary lipids and they are the main lipid metabolism tissues in the body [38]. Moreover, the liver is the main site for LC-PUFA synthesis [39]. These data indicated that lower levels of hepatic *Fads6* transcripts in carnivorous marine fish, like *T. ovatus*, may correlate with their limited LC-PUFA biosynthetic abilities [3].

In general, the mRNA levels of some genes in eukaryotic cells are dependent on transcription factors and RNA polymerases binding to specific sequences in gene promoters [40]. Consequently, the integrity and activity of a promoter can affect gene expression. Moreover, the transcription factor PPARα regulates lipid metabolism in mammals, and also influences transcription of the *Fads* gene family in fish and avian species [16,21,22,23]. Notably, evidence has suggested that *Fads2* promoter activity was increased by PPARα in fish and avian species [21,22]. Dual-luciferase reporter assays were conducted to clarify regulatory mechanisms, where PPARαb was believed to modulate *Fads6* expression. The analysis of truncated mutants indicated that *ToFads6* reporter activity was induced by the overexpression of PPARαb. The core binding region in the *ToFads6* promoter was −448 bp–+1 bp (Figure 5). This is the first evidence showing that the transcription of *ToFads6* can be upregulated by PPARαb. Furthermore, the deletion of the PPARαb M2 binding site (−113 bp to −87 bp) resulted in significantly reduced promoter activity (Figure 6), suggesting that the PPARαb M2 binding site was essential for *ToFads6* promoter activity.

To further confirm whether PPARαb was a transcription factor implicated in *ToFads6* function, the effects of PPARαb knockdown on *ToFads6* mRNA expression were investigated by qRT-PCR and western blotting in TOCF cells. These data showed that PPARαb upregulated both *ToFads6* mRNA and protein levels. Moreover, the PPARαb agonist (WY-14643) enhanced *ToFads6* transcription and consequently increased *ToFads6* mRNA, whereas PPARαb inhibition with GW6471, showed the opposite effects (Figure 7). Those results showed that both *Fads2* and *Fads6* expressions were controlled by PPARαb. The EMSA assay further demonstrated that PPARαb specifically bound to the *ToFads6* promoter at the M2 binding site (Figure 8).

**Figure 9:**
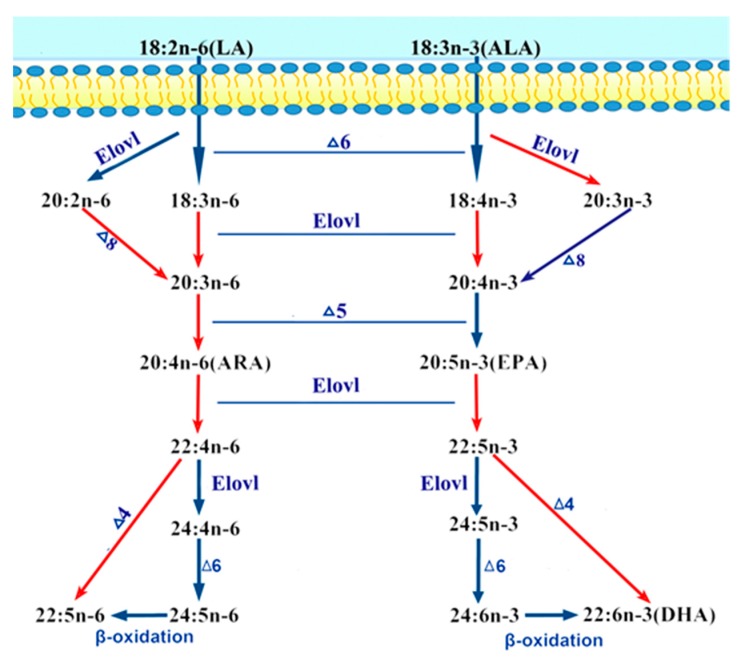
The proposed synthesis pathway of PUFA in *T. ovatus*. Red arrows represent the pathway confirmed in *T. ovatus* [27], blue arrows indicate the pathway confirmed in other marine fish [8,10,23].

In summary, the functional studies presented here have shown that *ToFads6* may effectively desaturate 22:4n-6 and 22:5n-3 substrates. Moreover, the proposed synthesis pathway of LC-PUFA was in *T. ovatus* (Figure 9). Furthermore, *ToFads6* expression was positively regulated by the transcription factor, PPARαb. The EMSA assays further showed that PPARαb bound effectively to the M2 binding site in the *ToFads6* promoter. Thus, a positive feedback mechanism, mediated by Fads6-induced PPARαb activation, is proposed in *T. ovatus*. PPARαb plays an important role in *ToFads6* regulation and may accelerate the biosynthesis of LC-PUFA through the upregulation of *ToFads6* transcription. These results provide new insights into the regulation and function of *Fads6* in fish and further reveal the complexity of associated regulatory mechanisms.

## 4. Materials and Methods

### 4.1. Ethics Statement

This study was performed in strict accordance with the guide for the Animal Care and Use Committee of South China Sea fisheries Research Institute, Chinese Academy of fishery Sciences (No.SCSFRI96-253, 5th sep 2015) in China.

### 4.2. Animals and Tissue Collection

*T. ovatus* juvenile fish (body weight: 62 ± 8.5 g) were collected from Linshui Marine Fish Farm in Hainan Province, China. The fish were raised on commercial feed (Hengxin, guangzhou, China, crude protein > 37%, crude fat > 7%) according to standard feeding schemes 2 weeks before sampling, and maintained in fresh seawater at 22 ± 1 °C, in dissolved oxygen > 6 mg/L, under a recirculating aquaculture system. For the study, fish tissue (*n* = 6) containing small intestine, liver, white muscle, brain, spleen, fin, gill, head kidney, stomach, blood, and male and female gonads were sampled, then flash frozen in liquid nitrogen, and stored at −80 °C until further use.

### 4.3. Gene Cloning and Bioinformatics of T. ovatus Fads6

Total RNA (1 μg) was extracted from *T. ovatus* liver by TRIzol Reagent (Takara, Kyoto, Japan). Subsequently, cDNA was synthesized using the Prime ScriptTM RT reagent Kit (Takara), according to the manufacturer′s instructions. According to genomic data of *T. ovatus* (Accession No. PRJEB22654 under the European Nucleotide Archive (ENA); Sequence Read Archive under BioProject PRJNA406847), a putative *ToFads6* sequence was obtained. To determine the veracity of the putative *Fads6* sequence, gene specific primers were designed (Table S1). The PCR protocol used was described previously [41]. The amplified products were purified using a DNA Purification Kit (Tiangen, Beijing, China), ligated into the pEASY-T1 vector (TransGen Biotech, China), and sequenced (Invitrogen, Shanghai, China). Validated plasmids were transformed into competent Trans1-T1 cells (TransGen Biotech, Beijing, China). A Blast search on the putative *Fads6* ORF sequence further confirmed accuracy and validity.

The deduced amino acid sequence of the cloned *ToFads6* open reading frame (ORF) was aligned with other *Fads6* ortholog ORFs from *Takifugu rubripes* (XP_003961115.2), *Tetraodon nigroviridis* (CAG03043.1), *Gasterosteus aculeatus* (ENSGACG00000014468), *Poecilia formosa* (XP_007570697.1), *Xiphophorus maculatus* (XP_005794734.1), *Oryzias latipes* (XP_023805434.1), *Oreochromis niloticus* (XP_005453754.1), *Danio rerio* (XP_003199708.1), *Astyanax mexicanus* (XP_022518693.1), *Lepisosteus oculatus* (XP_006635501.1), *Homo sapiens* (NP_835229.3), *Mus musculus* (NP_828874.3), *Gallus gallus* (XP_426241.2), and *Xenopus tropicalis* (XP_002939607.1). Multiple sequence alignments were conducted using ClustalX version 2.0 (Dublin, Ireland), with default parameters [42]. Phylogenetic analyses for all Fads6 amino acid sequences were performed using maximum likelihood (ML) methods (LG +G model, bootstrap 1000), with MEGA 6.0 [43]. All available *Fads6* genome sequences were downloaded from public databases: Ensembl (http://asia.ensembl.org/) and Genome Browser (http://genome.ucsc.edu/cgi-bin/hgBlat). The phylogenetic tree and genome structures were embellished using FigTree v1.4.2 (http://tree.bio.ed.ac.uk/software/figtree/) and Adobe PhotoShop CS6 (Adobe, San Jose, CA, USA).

### 4.4. Functional Characterization of the ToFads6 Desaturase

PCR products corresponding to the *ToFads6* ORF were amplified from *T. ovatus* liver cDNA using high fidelity Pfu DNA polymerase (Promega, Madison, Wis, USA) with primers incorporating *Hind* III and *Xho* I enzyme restriction sites (Table S1). The PCR products were digested with the above restriction endonucleases (Takara, Kyoto, Japan) and ligated into a similarly digested pYES2 yeast expression vector (Invitrogen, Shanghai, China). The recombinant plasmid (pYES2-Fads6) was transformed into *Saccharomyces cerevisiae*-competent cells (S.c. EasyComp Transformation Kit, Invitrogen). The selection of recombinant yeast colonies and subsequent yeast culture was prepared according to previously published methods [44,45]. Fatty acids (FA), designated as C18:2n-6 (Linoleic acid), C18:3n-3 (α-linolenic acid), C20:2n-6 (Eicosadienoic acid), C20:3n-6 (Dihomo-γ-linolenic acid), C22:4n-6 (Adrenic acid), and C20:5n-3 (Eicosapentaenoic acid, EPA) were used as substrates for detecting desaturase activity of *ToFads6*. The final concentrations of FA substrates varied according to their fatty acyl chain lengths: 0.5 mM (C18) and 0.75 mM (C20). Yeast cultures were incubated for two days at 30 °C, harvested, and washed twice, as described previously [8]. As a control, yeast were transformed with pYES2 only (no insert) and treated similarly. Fatty acid methyl esters (FAMEs) were prepared, extracted, purified and analyzed by thin-layer chromatography (TLC) and gas chromatography (GC2010-plus; Shimadzu, Kyoto, Japan) as described previously [46]. The proportion of substrate fatty acids converted to desaturated FA products was calculated as follows, (product area/(product area + substrate area) × 100 [8].

### 4.5. Real-Time Quantitative PCR Analysis

Specific primers for real-time quantitative PCR (qRT-PCR) were designed by Primer Premier 5.0 (Premier Biosoft, Palo Alto, CA, USA) based on cloned nucleotide sequences (Table S1). The translation elongation factor 1-alpha (*EF1α*) and PPARαb were verified by sequencing and were used as a reference and target gene, respectively [27,47]. The qRT-PCR amplifications were performed in a quantitative thermal cycler (Mastercycler EP Realplex, Eppendorf, Hamburg, Germany). The program parameters were 95 °C for 2 min, followed by 40 cycles of 95 °C for 10 s, 56 °C for 10 s, and 72 °C for 20 s. Amplification efficiencies of target and reference genes were observed from the slope of the log-linear portion of the calibration curve, with PCR efficiency = 10^(−1/Slope)^ − 1. Target gene expression levels were calculated using the 2^−ΔΔCt^ method [48].

### 4.6. Preparation of a Fads6 Polyclonal Antibody and Western Blotting Analysis

To prepare the polyclonal anti-Fads6 antibody, a specific domain (Fads6 ^aa257–380^) of *Fads6* was amplified using gene-specific primers (Table S1). The resulting PCR product was inserted into the pET-B2M vector using *Nde* I/*Xho* I sites. To express recombinant *T. ovatus* Fads6 protein (rToFads6), the recombinant plasmid was transformed into *Escherichia coli* BL21 (DE3) (Novagen, Darmstadt, Germany). The rToFads6 was purified as described previously [49]. To generate a polyclonal antibody, purified rToFads6 protein was injected into white New Zealand rabbits using standard methods [50]. Once generated, the polyclonal antibody was pre-adsorbed using *E. coli* lysate supernatants to eliminate inhomogeneous antibodies and depurated on a HiTrapTM Protein A HP column on a AKTAprime™ Plus system (GE Healthcare, Boston, MA, USA).

To confirm specificity of the rabbit anti-Fads6 antibody, human embryonic kidney (HEK293T) cells were transfected with pcDNA3.1 and pcDNA3.1-Fads6 for 48 h. After this period, cells were harvested by centrifugation at 160× *g* for 10 min at 4 °C. Total protein was extracted using ProteoPrep^®^ Total Extraction Sample Kit (Sigma-Aldrich, St. Louis, Mo, USA). This protein was electrophoresed on 12% SDS-PAGE and electrophoretically transferred to polyvinylidene fluoride (PVDF) membranes (Millipore, Billerica, MA, USA) using the PierceG2 Fast Blotter (25 V for 10 min, Pierce, Rockford, IL, USA). Western blotting analyses were executed according to a previously described protocol [51].

To observe endogenous Fads6 expression, TOCF cells were transfected with PPARαb siRNA, cells were harvested and lysed as described previously [27,52]. Total protein was incubated with/without calf intestinal alkaline phosphatase (CIAP) (20 U) at 37 °C for 30 min, separated by 12% SDS-PAGE and transferred to PVDF membranes using the PierceG2 Fast Blotter. Primary antibodies, anti-Fads6, murine anti-Flag (Sigma-Aldrich, St. Louis, MO, USA), the loading control (1;1000), and the anti-glyceraldehyde 3-phosphate dehydrogenase antibody (GAPDH; Sigma-Aldrich) were incubated with the PVDF membrane in 1% (*w*/*v*) nonfat milk in Tris-buffered saline and Tween 20 (TBST) buffer (0.1% Tween 20) for 3 h. Horse radish peroxidase-(HRP)-conjugated goat anti-rabbit antibody (1:3000) was used as a secondary antibody (Sigma-Aldrich). The results were observed using an electrochemiluminescence (ECL) system (Tanon, Shanghai, China).

### 4.7. Cloning of the Fads6 Promoter and Construction of Deletion Mutants

Genomic DNA was extracted from the muscle tissue of *T. ovatus*, as described previously [53], and used as a template for candidate promoter cloning. The sequence upstream of the *Fads6* gene was obtained from genomic sequencing data of *T. ovatus*. To identify the core promoter region of *ToFads6*, the forward primers (Fads6-p1F, Fads6-p2F, Fads6-p3F, Fads6-p4F, and Fads6-p5F), were designed with a 5′ *Hind* III site, and the common reverse primer (Fads6-pR) was designed with a 3′ *Xho* I site (Table S1). These primers were used to obtain a full-length promoter fragment (Fads6-p1, 2372 base pairs (bp)) and four truncated fragments: (i) Fads6-p2, 1925 bp; (ii) Fads6-p3, 1370 bp; (iii) Fads6-p4, 759 bp; and (iv) Fads6-p5, 448 bp (Figure 5). The truncated mutants were amplified using PrimeSTAR Master Mix (Takara, Japan). The program parameters were 95 °C for 4 min, followed by 30 cycles of 95 °C for 40 s, 56 °C for 40 s, and 72 °C for 1 min. A general DNA Purification Kit (Tiangen, China) was used to purify the PCR products. All purified PCR products and a pGL3-basic (Promega, USA) vector were digested with *Hind* III and *Xho* I, and concentrated by T4 DNA ligase (Takara, Japan) overnight at 16 °C. Recombinant plasmids were extracted using EndoFree Plasmid Giga Kit (Tiangen, China), and constructs were confirmed by sequencing, as described above.

### 4.8. Construction of Truncated Mutants for the Identification of Predicted Transcription Factor (TF) Binding Sites in the Fads6 Promoter

To determine the potential function of PPARαb binding sites on the core Fads6 promoter, four truncated mutations were generated. The transcription factor binding site prediction (TFBS)-JASPAR database (http://jaspar.genereg.net/), TRANSFAC^®^, and MatInspector^®^ were used to search for potential binding sites with PPARαb, in the Fads6 promoter sequence. According to the manufacturer′s protocol, truncated mutants were designed and produced with Muta-directTM site-directed mutagenesis kit (SBS Genetech, Shanghai, China) from the deletion mutant pGL3-basic-Fads6-5, which was wild-type. The predicted four binding sites (M1, M2, M3, and M4) were deleted, and the corresponding transcription factor (TF) binding site sequences are shown in Figure 6A. Furthermore, to acquire TF binding site mutations, the method of PCR augmentation referred to a previous study [54]. The influence of TF binding site mutations on promoter activity of ToFads6 were determined by Dual luciferase assay.

### 4.9. Cell Culture, Transfection, and Luciferase Assay

HEK293T cells were cultured in DMEM (Gibco, Grand Island, NY, USA), supplemented with 10% fetal bovine serum (FBS) (Invitrogen, USA), 100 U mL^−1^ penicillin, and 100 μg mL^−1^ streptomycin at 37 °C in a humidified incubator under 5% CO_2_. Transfection and dual luciferase reporter assays were described by Li et al. [55]. Relative luciferase activities (firefly and renilla luciferase activities) were measured by the VICTOR^TM^ X2 Multilabel Plate Reader (PerkinElmer, Inc., Waltham, MA, USA). Moreover, small interfering RNA (siRNA) or plasmids were transfected using Lipofectamine RNAiMAX or Lipofectamine 2000 transfection reagent (Invitrogen, USA) into TOCF cells according to manufacturer′s instructions [27,52].

### 4.10. Expression Analysis of ToFads6 with ToPPARαb

Construction of ToPPARαb recombinant plasmids and siRNA was described as by Zhu et al. [27]. The PPARαb siRNA sequence is listed in Table S1. After transfection into TOCF cells, total RNA and protein were isolated at specific time points (0 h, 6 h, 12 h, and 24 h), as described above. Additionally, agonists and transcription factor inhibitors were used to investigate the role of transcription factors on the regulation of *Fads6* desaturase in *T. ovatus*. WY-14643 (Sigma-Aldrich) was used as a PPARαb agonist, whereas GW6471 (Sigma-Aldrich) was used as a PPARαb inhibitor. TOCF cells were seeded in 6-well plates, at a density of 2 × 10^6^ cells per well, in L15 media supplemented with 10% FBS. Plates were incubated for 24 h, at 28 °C. The cells were washed and incubated for 1 h in FBS-free L15 medium, prior to incubation with WY-14643 (0.1, 1, and 4 μmol/L) or GW6471 (0.1, 1, and 4 μmol/L), in triplicate for 24 h. Cells were lysed in the wells and harvested for RNA extraction after 24 h.

### 4.11. Electrophoretic Mobility Shift Assay (EMSA)

EMSA was carried out as previously described [56]. In brief, the lysates of HEK293T cells, transfected with pcDNA3.1-Flag-PPARαb were prepared for DNA/protein conjugation reactions. The EMSA Probe Biotin Labeling Kit (Beyotime, Shanghai, China) was used to label mutated and wild-type oligonucleotides (Table S1) for EMSA biotin-labeled probes, according to the manufacturer′s instructions. DNA/protein binding reactions were performed using an EMSA/Gel-Shift Kit (Beyotime, China) at 25 °C, based on the manufacturer′s instructions. To detect the specificity of DNA/protein binding reactions, competition assays were carried out with 100 × excessive unlabeled wild-type or mutated probes. Completed reactions were separated on nondenaturing 4% PAGE gels for 20 min. The proteins were developed by autoradiography using a LightShift^®^ Chemiluminescent EMSA Kit (Pierce, USA).

### 4.12. Statistical Analysis

All experiments were carried out in triplicate. All values were expressed as the mean ± SD (*n* = 3). Significant differences were analyzed using one way analysis of variance tests. *p* < 0.05 was considered statistically significant.

## Figures and Tables

**Table 1:** Conversion rates of pYES2-Fads6 transformed yeast grown in C18:2n-6, C18:3n-3, C20:2n-6, C20:3n-6, C22:5n-3, and C22:4n-6 substrates.

FA Substrate	Product	Conversion (%)	Activity
C18:2n-6	C18:3n-6	0	Δ6
C18:3n-3	C18:4n-3	0	Δ6
C20:2n-6	C20:3n-6	0.17 ± 0.02	Δ8
C20:3n-6	C20:4n-6	0.54 ± 0.06	Δ5
C22:5n-3	C22:6n-3	39.29 ± 4.4	Δ4
C22:4n-6	C22:5n-6	1.04 ± 0.11	Δ4

Conversions are expressed as a percentage of total FA substrate converted to desaturated products.

**Table 2:** Primers used for site-directed mutagenesis of putative binding sites in the *ToFads6* promoter.

Putative Binding Sites	Nucleotide Sequence	Mutation
M1	5′ CTGGACTGCATCAAGAGTCA 3′	deletion
M2	5′ ATGCTAACTTTGACCTGTGGACTGTGC 3′	deletion
M3	5′ AGCAGCTGCAGTTTTGTGTG 3′	deletion
M4	5′ GGGGGGAACAGAGGAGAGG 3′	deletion

## Supplementary Materials

Supplementary materials can be found at http://www.mdpi.com/1422-0067/20/1/23/s1.

## Abbreviations:

Fads	Fatty acid desaturases
EMSA	Electrophoretic mobile shift assays
LC-PUFA	Long-chain polyunsaturated fatty acids
PPARαb	Transcription factor peroxisome proliferator-activated receptor alpha b
Elovl	Elongation of very long-chain fatty acids
HNF4	Hepatocyte nuclear factor-4
NR1H3	Nuclear receptor subfamily 1 group H member 3
SREBP	Sterol-regulatory element binding protein
NF-κB	Nuclear factor-kappa B
PPREs	PPAR response elements
FAMEs	Fatty acid methyl esters
TLC	Thin-layer chromatography
TOCF	T. ovatus caudal fin
