# Supplementary material for: Identification of Fatty Acid Desaturase 6 in Golden Pompano Trachinotus Ovatus (Linnaeus 1758) and Its Regulation by the PPARαb Transcription Factor
Source: Int J Mol Sci. 2018 Dec 21;20(1):23. doi: 10.3390/ijms20010023 (PMC6337163; doi:10.3390/ijms20010023)
Supplement: Supplementary file 1 [file ijms-20-00023-s001.pdf]

Supplementary Materials:

```
1 GGCTGGGCTTCTCTGACAATGCTTCAAACCAATAAGGACTTTCGCAATGCTAACTTTGACCTGTGGACTGTGCAGCATTTAGCACCAGCT 90
91 GCCTGCAAGAGGACACAACCTGCACGGTGACTTTTTGTTTTGAGCCGGTTCATCAGCAGAGGCAACTGACatgcagaatgtaccggagga 180
1 M Q N V P E E 7
181 gtggagggaaggacgcagtgagagcaggggggggatgttagtcaagaggaggggtgattcaggagagcgcagcagtggagacatgaaggg 270
8 W R E G R S E S R G G M L V K R R G D S G E R S S G D M K G 37
271 agagtggaggaagaatgcagggacaggtggggaagaggggagaaagagacgctgatgatggagctgacgaggctggtgcagacgatggt 360
38 E W R K N A G T G G E E G E K E T L M M E L T R L V Q T M V 67
361 gaaggagagcagctggtgggagaggagggggatcgactgcagcatcctggcagcagcattcctctgtctgcctccggccttcctgctgct 450
68 K E S S W W E R R G I D C S I L A A A F L C L P P A F L L L 97
451 gtcctcctctcagatcctgtggttttcggcgggcatgctgctgatgggcgtagctcacgctgtcatcaccatcaaaggacacatctggc 540
98 S S S Q I L W F S A G M L L M G V A H A V I T I K G T H L A 127
541 cagccacggggcgctgagtgagtcacaggcctgggggaagttctgggccgtcttcttcatcgaggtgtgtggctcgttctcggcgcgggc 630
128 S H G A L S E S Q A W G K F W A V F F I E V C G S F S A R A 157
631 cggcgtgcagggacacattaagatgcatcatgctcatactaatgtcattggactgggcgactccagcgtgtggaaggtccccttcctgcc 720
158 G V Q G H I K M H H A H T N V I G L G D S S V W K V P F L P 187
721 tcgcactgtctacctgttcttagccccccctggccgtacccatcatcactccgcttgttgcactcgctcatctcaaaggacattcctcggc 810
188 R T V Y L F L A P L A V P I I T P L V A L A H L K G H S S A 217
811 ccacatcgtcaggaccatcctgatggtgacgctgggtctgtattctcagtactggctgctgatccacgtctccgggttcaagtcccccct 900
218 H I V R T I L M V T L G L Y S Q Y W L L I H V S G F K S P L 247
901 cagcaccttgctctgcatgctcatctgcagagcaatgttctctgtgccatacatccatgtcaacattttccagcacatcggcctccccat 990
248 S T L L C M L I C R A M F S V P Y I H V N I F Q H I G L P M 277
991 gttctccccgacccgtcgaccaaagaggatctaccagatgacccacggagtcctgaacctgccccggaaccctgtgctagactggacctt 1080
278 F S P T R R P K R I Y Q M T H G V L N L P R N P V L D W T F 307
1081 tggacactcacttatcaactgccacgtggagcaccatctgttccccttcctgtccgataacatgtgcttaaaggtgaagcctgtcgtgtc 1170
308 G H S L I N C H V E H H L F P F L S D N M C L K V K P V V S 337
1171 caagtatctgactgaaaaacagcttccataccaggaggacagctacctctctcgcctgaacttcttcttccacagataccaggagctgat 1260
338 K Y L T E K Q L P Y Q E D S Y L S R L N F F F H R Y Q E L M 367
1261 ggtgttcgctcctcccatcacagagctggtgggggtgcagtgaTGACCATGAACTGTTCAGACTAGTGACTGCTGCTGCTGCTGTTCAAA 1350
368 V F A P P I T E L V G V Q * 380
1351 AAGCACAAGCTTCAGGGTTTTAAGTGCATTTTCTGCTGTCATGCTACAACCTCCACGGTC
```

Figure S1. Sequence of cDNA and deduced protein of ToFads6.

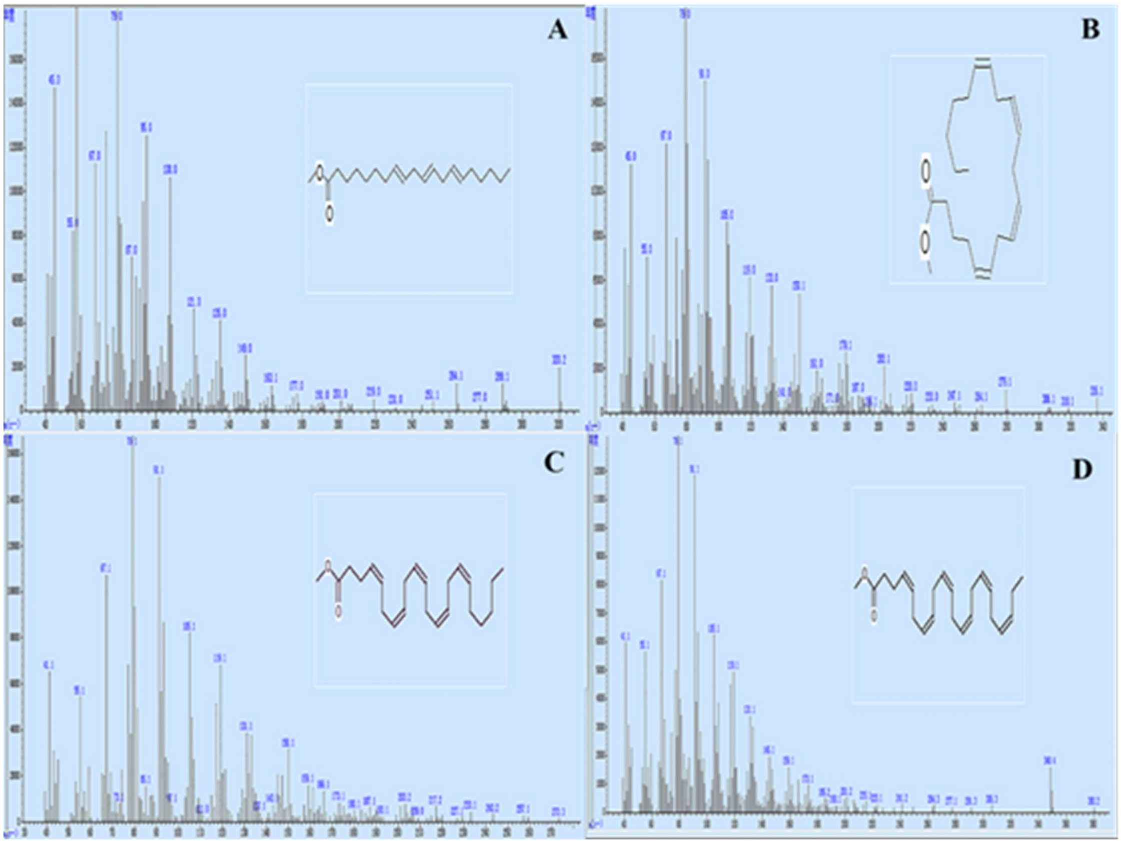

Figure S2. Fatty acid profiles determined by Gas chromatograph-mass spectrometry (GC-MS) analysis. Picolinyl esters were prepared from FAME extracted from yeast transformed with pYfads6 and grown in the presence of C18:3n-3, C18:2n-6, C20:2n-6, C20:3n-6, C22:4n-6, and C22:5n-3. The identities of the peaks were only confirmed as C20:3n-6 (A),C20:4n-6 (B), C22:5n-6 (C) and C22:6n-3 (D). Vertical axis, % abundance; horizontal axis, mass-to-charge (m/z) ratio.

Table S1. Sequences of primers used for this study.

Subject and Primers	Nucleotide sequence	Fragment (bp)
**Primers for sequence cloning**		
Fads6-ORF-F1	GCTGGGCTTCTCTGACAA	826
Fads6-ORF-R1	TGGTCCTGACGATGTGGG	
Fads6-ORF-F2	CTCGCACTGTCTACCTGTT	691
Fads6-ORF-R2	GACCGTGGAGGTTGTAGC	
Fads6-pYES2-F	CCCAAGCTTATGCAGAATGTACCGGAGGA	1143
Fads6-pYES2-R	CCGCTCGAGTCACTGCACCCCCACCAGCTC	
**Deletion mutant construction**		
Fads6-p1F	CCCAAGCTTCAGCTCAGTCTCATATAAT	2372
Fads6-p2F	CCCAAGCTTAAACACTGTTGCAATAGCA	1925
Fads6-p3F	CCCAAGCTTTTACTTTGGAGATTCAGAT	1370
Fads6-p4F	CCCAAGCTTGCATGGCCTCGAGCCCAGAAC	759
Fads6-p5F	CCCAAGCTTGGGGGTGAACGACATGCAGAA	448
Fads6-pR	CCCCTCGAGGTCAGTTGCCTCTGCTGATGA	
**Primers for qRT-PCR**		
qRT-F6-F	TACCAGATGACCCACGGA	214
qRT-F6-R	AGAAGTTCAGGCGAGAGAG	

qRT-PPARαb-F	AGTGACCTGGCTCTGTTTG	145
qRT-PPARαb-R	AAGCGTCGTCTGGATGATT	
EF1α-F	AAGCCAGGTATGGTTGTCAACTTT	189
EF1α-R	CGTGGTGCATCTCCACAGACT	
**Primers for antibody**		
F6-AF	AGAGCAATGTTCTCTGTGCC	372
F6-AR	TCACTGCACCCCCACCAGCTC	
**TF ORF cloning**		
PPARαb-F	CTAGCTAGCATGGTCGACATGGAGAGCCAC	1407
PPARαb-R	CCCAAGCTTTCAGTACATGTCTCTGTA	
**siRNA**		
PPARαb-si	GCGAATGCAGGAGAGCATT	
PPARαb-NC	ACGUGACACGUUCGGAGAATT	
**EMSA assays**		
Fads6-MT	ACCTGGGTCAACTGGCTGAACACGTAG	
Fads6-WT	ATGCTAACTTTGACCTGTGGACTGTGC	

Table S2. Lengths of exons and introns of each *Fads6* gene.

	Exon1	Intron1	Exon2	Intron2	Exon3	Intron3	Exon4	Intron4	Exon5	Intron5	Exon6	Intron6	Exon7
Trachinotus ovatus	279	2302	168	364	180	208	186	1999	183	1153	147	434	126
Gasterosteus aculeatus	262	700	168	656	180	108	186	582	183	233	137		
Oreochromis niloticus	242	2966	168	2359	180	176	186	1917	183	190	147		
Oryzias latipes	393	1692	168	1588	180	97	189	856	183	239	144		
Poecilia formosa	258	3282	168	1498	180	619	186	2400	183	521	144		
Xiphophorus maculatus	255	2152	168	1474	180	541	186	3030	183	529	147		
Takifugu rubripes	249	1064	168	94	177	148	189	780	183	71	144		
Tetraodon nigroviridis	114	576	153	289	165	161	177	152	192	926	183	71	144
Danio rerio	201	7251	168	2435	180	1012	183	4081	183	3298	147		
Astyanax mexicanus	207	1723	168	1824	180	1376	180	3086	225	-	-		
Lepisosteus oculatus	384	1015	168	838	180	549	186	2218	183	158	144		
Xenopus tropicalis	330	120	168	1440	180	208	180	88	183	546	147		
Gallus gallus	351	810	168	193	180	589	186	382	183	597	147		
Homo sapiens	240	662	171	9812	180	1274	186	1484	183	926	144		
Mus musculus	159	609	171	10239	180	631	186	919	183	544	147		

Table S3. Fads6 proteins used in multiple alignment.

Species	Protein name	GenBank No.
Trachinotus ovatus	Fads6	MG674450
Takifugu rubripes	Fads6	XP_003961115.2
Gasterosteus aculeatus	Fads6	ENSGACG00000014468
Oreochromis niloticus	Fads6	XP_005453754.1
Danio rerio	Fads6	XP_003199708.1
Homo sapiens	Fads6	NP_835229.3
